# A Multienzyme Complex Channels Substrates and Electrons through Acetyl-CoA and Methane Biosynthesis Pathways in *Methanosarcina*

**DOI:** 10.1371/journal.pone.0107563

**Published:** 2014-09-18

**Authors:** Dillon J. Lieber, Jennifer Catlett, Nandu Madayiputhiya, Renu Nandakumar, Madeline M. Lopez, William W. Metcalf, Nicole R. Buan

**Affiliations:** 1 Department of Biochemistry and Redox Biology Center, University of Nebraska-Lincoln, Lincoln, Nebraska, United States of America; 2 Redox Biology Center Metabolomics Core Facility, University of Nebraska-Lincoln, Lincoln, Nebraska, United States of America; 3 Department of Microbiology, University of Illinois at Urbana-Champaign, Urbana, Illinois, United States of America; Laurentian University, Canada

## Abstract

Multienzyme complexes catalyze important metabolic reactions in many organisms, but little is known about the complexes involved in biological methane production (methanogenesis). A crosslinking-mass spectrometry (XL-MS) strategy was employed to identify proteins associated with coenzyme M-coenzyme B heterodisulfide reductase (Hdr), an essential enzyme in all methane-producing archaea (methanogens). In *Methanosarcina acetivorans*, Hdr forms a multienzyme complex with acetyl-CoA decarbonylase synthase (ACDS), and F_420_-dependent methylene-H_4_MPT reductase (Mer). ACDS is essential for production of acetyl-CoA during growth on methanol, or for methanogenesis from acetate, whereas Mer is essential for methanogenesis from all substrates. Existence of a Hdr:ACDS:Mer complex is consistent with growth phenotypes of ACDS and Mer mutant strains in which the complex samples the redox status of electron carriers and directs carbon flux to acetyl-CoA or methanogenesis. We propose the Hdr:ACDS:Mer complex comprises a special class of multienzyme redox complex which functions as a “biological router” that physically links methanogenesis and acetyl-CoA biosynthesis pathways.

## Introduction

Multienzyme complexes catalyze important reactions in central metabolic processes such as photosynthesis, respiration, and amino acid synthesis. We wanted to determine whether multienzyme complexes are also involved in the central metabolic process of biological methane production (methanogenesis) in methane-producing archaea (methanogens). Methanogens are obligately anaerobic archaea that derive all their energy for growth by reducing carbon sources such as acetate, formate, CO_2_, methanol, methylamines and methyl-sulfides to methane gas. Metabolic engineering of methanogens is an attractive prospect for increasing the yield and rate of renewable methane production from biomass in anaerobic digesters. However, successful metabolic engineering requires not only an in-depth understanding of methanogen physiology, but also a knowledge of which reactions are physically linked by multienzyme complexes. A detailed, three-dimensional spatial model of methanogenesis proteins would be useful in these efforts.

**Figure 1:**
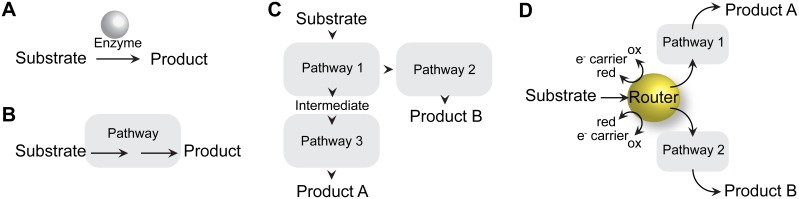
Organization of cellular metabolism. Metabolic reactions in a cell can be catalyzed by *A*, individual enzymes, or *B*, multienzyme complexes that channel substrates and/or sequester intermediates in a pathway. Pathways in the cell can be connected in series, *C*, or in parallel by *D*, metabolic “routers” that channel electrons and substrates to either of two metabolic pathways.

Metabolic reactions that are linked by multienzyme complexes have clear advantages over reactions that are catalyzed by individual, unlinked enzymes [1]. Complexes channel substrates to prevent diffusion of intermediates into bulk cytoplasm, effectively increasing the relative local concentration of reactants in subsequent pathway steps, speeding the overall rate of production of the final product, and preventing diffusion of toxic intermediates that can damage cell constituents. Complexes can also provide a means of co-regulating pathway enzymes or ensuring proper enzyme dosage (Figure 1). Methanogens obtain up to 1 mole ATP per mole substrate consumed and live near the thermodynamic lower limit of life [2]. Substrate channeling via multienzyme complexes would provide a kinetic advantage by ensuring maximal efficiency for converting substrate to ATP generation. We used in vivo crosslinking, tandem affinity purification, and peptide mass spectrometry (XL-MS) to look for complex formation among methanogenesis enzymes. XL-MS is a reliable technique for identifying protein:protein interactions by identifying crosslinked partners which elute together after affinity column purification. A recent effort in *Saccharomyces cerevisiae* has successfully demonstrated the ability to use XL-MS to reproduce 30 years of protein:protein interaction data and to predict new interactions which were subsequently verified genetically [3]. Though commonly applied to the study of cell signaling networks, we surmised that XL-MS is a valuable technique for identifying protein:protein interactions between methanogenesis enzymes and electron transfer proteins in the methanogen, *Methanosarcina acetivorans*.

**Figure 2:**
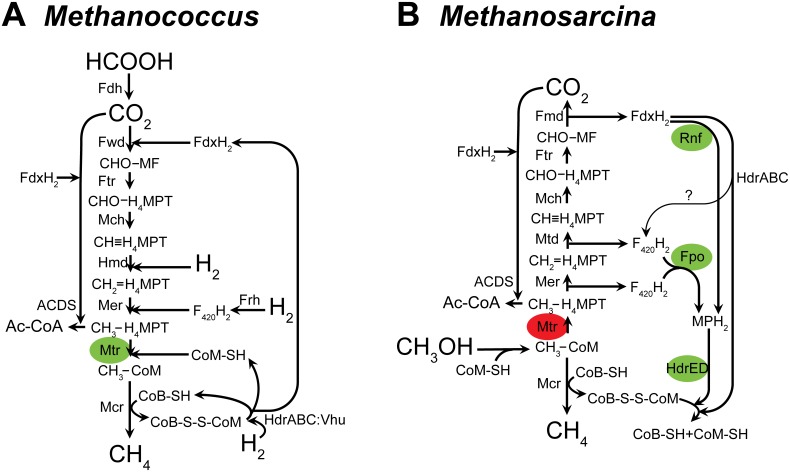
Comparison of methanogenesis pathways. *A*, Hydrogenotrophic methanogenesis in *Methanococcus maripaludis. B*, Methylotrophic methanogenesis in *Methanosarcina acetivorans*. Green ovals: energy-conserving reactions. Red ovals: energy-consuming reaction. Please see text for abbreviations.

The majority of cultivated methanogen strains are restricted to using formate or CO_2_ as the sole carbon source, and these methanogens use the hydrogenotrophic methanogenic pathway, which relies on reducing equivalents from hydrogen gas to reduce formate or CO_2_ to methane (Figure 2) [4]. Methanogens which solely utilize the hydrogenotrophic pathway have electron transport systems that are different from the electron transport systems of generalist methanogen species like *Methanosarcina acetivorans*. The generalist organism *M. acetivorans* is capable of using the methylotrophic (methanol, methylamines, methylsulfides), carboxidotrophic (CO), and the acetoclastic pathways, but cannot use the hydrogenotrophic or methyl respiration pathways due to the lack of expression of suitable hydrogenases [5], [6], [7], [8].

We wanted to identify proteins that form complexes with coenzyme M-coenzyme B (CoM-S-S-CoB) heterodisulfide reductase (HdrED) in *M. acetivorans*. HdrED is essential for methylotrophic and aceticlastic growth and is likely to participate in protein:protein interactions with other enzymes of the methanogenesis pathway in *Methanosarcina*. Previous reports showed that CO oxidation can be coupled to CoM-S-S-CoB reduction in cell extracts in *Methanosarcina barkeri* MS. The system was then reconstituted using pure Hdr and CO dehydrogenase (a subcomplex of ACDS enzyme) components from *Methanosarcina thermophila* [9], [10]. The CO:Hdr activity in both reports required the addition of ferredoxin and membranes. XL-MS would seem to be a suitable technique to address whether the CO:Hdr complex occurs in vivo. In this work we have identified proteins that co-purify with the HdrD1 subunit, which contains the CoM-S-S-CoB reductase active site. We report that the proteins with the highest confidence for interacting with HdrD1 are the β subunit of acetyl-CoA decarbonylase/synthase (ACDS), and methylene-tetrahydromethanopterin reductase (Mer).

## Results

### Strep-tagged HdrD1 protein forms high molecular-weight complexes in vivo

**Figure 3:**
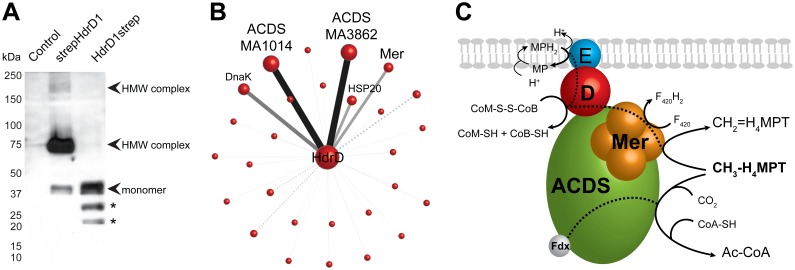
XL-MS identification of a multienzyme complex in *Methanosarcina*. *A*, Detection of HdrD complexes. 2 µg crosslinked cell lysates from controls or strains expressing strep-tagged HdrD1 protein were analyzed by Western blot. Arrows indicate the position of strep-tagged HdrD1 monomer and crosslinked high molecular weight (HMW) complexes. * = degraded HdrD1strep protein. *B*, HdrD co-purified proteins detected by mass spectrometry. Node sizes, line opacity and line widths are proportional to the average peptide hit score of the protein detected in biological duplicates. Dotted lines denote an average score below 100, solid lines denote an average score of 100 and above. Image created with Cytoscape [36]. *C*, Putative model of the Hdr:ACDS:Mer complex. During methylotrophic growth, both ACDS and Mer use methyl-H_4_MPT as a substrate. Black dotted lines = electron flow between active sites. HdrE (blue) or HdrD (red), Mer is a tetramer (orange), and ACDS is composed of 5 subunits in a (α_2_ε_2_)_4_β_8_(γδ)_8_ configuration (green) [13], [16], [37].

The HdrD1 protein was chosen for XL-MS experiments because it contains the CoM-S-S-CoB heterodisulfide reductase active site and the gene has been shown to be essential for growth on trimethylamine, methanol, methanol + acetate, and acetate [11]. Plasmids expressing N- or C-terminal strep-tagged HdrD1 protein (pNB636 and pNB637, respectively) were recombined onto the *M. acetivorans* chromosome (Table 1). A StrepTagII peptide affinity tag (WSHPQFEK) was chosen instead of a 6XHistidine tag because His tags have the potential to interfere with assembly of metal clusters. The covalent crosslinker, dimethylsuberimidate (DMS), was added to cells before protein purification to stabilize high molecular weight complexes. DMS is an 11 Å amide crosslinker that crosses cell membranes and is unaffected by residual sulfide present in methanogen cell preparations. N-terminally tagged HdrD1 protein (strepHdrD1) was stably expressed as judged by Western blot (Figure 3a). strepHdrD1 appeared to form two high-molecular weight complexes, observed as bands of approximately 75 kDa and 150 kDa in addition to a band corresponding to strepHdrD1 monomer (45 kDa). C-terminally tagged HdrD1strep protein was not stably expressed and was therefore not used for further experiments (Figure 3a). Instability of C-terminally tagged HdrD1strep protein suggests the 8-amino acid strep tag interferes with correct protein folding.

**Table 1:** Plasmids and strains used in this study.

Plasmids and *E. coli* strains				
NB #	Genotype	Purpose	Plasmid name	Reference
**pJK026A derivatives**				[18], [36]
139	*P_mcrB_strephdrD1*	Constitutive production of strepHdrD1protein in *Methanosarcina*	pNB636	This study
140	*P_mcrB_hdrD1strep*	Constitutive production of HdrD1strepprotein in *Methanosarcina*	pNB637	This study
145	*P_mcrB_strephdrD2*	Constitutive production of strepHdrD2protein in *Methanosarcina*	pNB661	This study
146	*P_mcrB_hdrD2strep*	Constitutive production of HdrD2strepprotein in *Methanosarcina*	pNB662	This study
***Methanosarcina acetivorans*** ** C2A strains**				
**NB #**	**Genotype**	**Purpose**	**Reference**	
95	*Δhpt::P_mcrB_tetR*/φC31*int/attP*	Parental strain	same as WWM74 [18]	
75	*Δhpt::P_mcrB_tetR*/φC31*int/att* pJK026A	control for protein overexpression andnon-specific binding to streptavidinagarose resin	This study	
79	*Δhpt::P_mcrB_tetR*/φC31*int/att* pNB636	*_strep_hdrD1* constitutive overexpressionfrom *hpt* locus under *P_mcrB_* promoter	This study	
80	*Δhpt::P_mcrB_tetR*/φC31*int/att* pNB637	*hdrD1_strep_* constitutive overexpressionfrom *hpt* locus under *P_mcrB_* promoter	This study	
41	*Δhpt::P_mcrB_tetR*/φC31*int/att* pNB661	*_strep_hdrD2* constitutive overexpressionfrom *hpt* locus under *P_mcrB_* promoter	This study	
42	*Δhpt::P_mcrB_tetR*/φC31*int/att* pNB662	*hdrD2_strep_* constitutive overexpressionfrom *hpt* locus under *P_mcrB_* promoter	This study	

### HdrD1 interacts with an ACDS:Mer complex

In order to identify constituents of the crosslinked strepHdrD1 complex, we used affinity purification and peptide mass spectrometry. Peptide masses from biological duplicate samples were compared to predicted mass database of *M. acetivorans* C2A to identify proteins contained in the eluate (Tables S1–S4 in File S1). Protein samples from mock co-purifications conducted with cells expressing β-glucoronidase were used as a control to screen for nonspecific binding to the resin. Sixteen proteins with significant scores were detected from duplicate control samples (Figure S1). After subtracting these nonspecific proteins from the list of proteins that co-purified with strepHdrD1, 29 proteins with significant scores (>100) remained. Of these 29 proteins, the highest score was for HdrD1, as would be expected (Table 2). The second-highest corresponded to the CdhC subunit of acetyl-CoA decarbonylase synthase (ACDS enzyme). CdhC protein is the β subunit of ACDS enzyme and houses a NiFeS “A site” responsible for cleaving acetyl-CoA during growth on acetate or for creating acetyl-CoA from CO_2_ and CH_3_-H_4_MPT during growth on methanol [12], [13], [14]. Mer was also detected in the co-purified samples (Table 2). During growth on methanol, Mer catalyzes the F_420_-dependent oxidation of CH_3_-H_4_MPT to CH_2_-H4MPT in the oxidative branch of the methylotrophic methanogenesis pathway [15], [16]. The chaperones DnaK and Hsp20 also co-purified with strepHdrD1, suggesting that overexpression may have taxed the protein folding machinery of the cell, an unsurprising result considering that strepHdrD1 expression is driven by the *P_mcrB_* promoter, which has the highest expression level in methanogens [17], [18], [19]. The remaining 23 proteins had scores less than 100, suggesting that they are minor constituents of an HdrD1 complex (Figure 3b).

**Table 2:** HdrD1 protein:protein interactions detected by Mass Spectrometry^a^.

Protein gi#	Gene MA#	Function	Average score^b^
20089573	MA0688	HdrD1, heterodisulfide reductase, subunit D	432
20089889	MA1014	CdhC, acetyl-CoA decarbonylase/synthase complex subunit beta	383
20092658	MA3862	CdhC, acetyl-CoA decarbonylase/synthase complex subunit beta	383
20090337	MA1478	molecular chaperone DnaK	220
20092530	MA3733	Mer, methylenetetrahydromethanopterin reductase	153
20093358	MA4574	hsp20/alpha crystallin family protein	151

a Proteins also detected in the control samples have been omitted.

b Proteins were identified in duplicate biological samples.

While ACDS, Mer, and molecular chaperones are proteins one would expect are present in high abundance, not all high-abundance proteins co-purified with strepHdrD1. For instance, Methyl-CoM reductase, Mcr, the protein of highest abundance in methanogen cells, was not detected in strep HdrD1 eluates, and none of the other methanogenesis proteins were detected. The highest peptide hits in the control samples were elongation factor EF-2, MtaC1 (methanol-5-hydroxybenzimidazolylcobamide co-methyltransferase, isozyme 1), glutamate-ammonia ligase, and Hsp60. The high peptide hit scores of ACDS and Mer in biological replicates indicates that the co-purification procedure and control screen was sufficiently stringent.

We anticipated that HdrE should be identified. HdrE is an integral membrane *b*-type cytochrome that delivers electrons to HdrD1. HdrE was identified by purification of CoM-S-S-CoB reductase activity from the membrane fraction of *Methanosarcina barkeri*. Therefore HdrED interaction was expected to be robust during purification. Batch resin binding with whole cell lysate was used to improve the probability of capturing membrane protein partners, and the main HMW complex band visible in the Western blot corresponds to the expected size of the HdrE:strepHdrD1 crosslinked species (∼75 kDa), suggesting that the HdrE:strepHdrD1 complex should have been detected. It is possible that despite utilization of crosslinker and batch binding of whole lysate to the streptactin resin, HdrE may not have been detectable by mass spectrometry due to problems with complete digestion of membrane proteins or the solubility of hydrophobic peptides. Therefore it is possible that HdrE, along with other highly hydrophobic proteins, may have been missed.

### HdrD2 does not form high-molecular-weight complexes

*M. acetivorans* has a close homolog of HdrD1, HdrD2, which is encoded by gene MA0526. HdrD2 is 31% identical, 47% similar to HdrD1 by primary amino acid sequence. Unlike HdrD1, expression of HdrD2 is not essential for growth [11]. However, we hypothesized that HdrD1 and HdrD2 may share overlapping cellular functions because of their similar sequences. When strep-tagged HdrD2 is expressed in *M. acetivorans*, we could not detect a high-molecular-weight complex after crosslinking (data not shown). We also did not detect interacting proteins with a MASCOT score above 100 (Table S5 in File S1). These results indicate that although HdrD1 and HdrD2 share significant amino acid sequence similarity, they do not crosslink with the same proteins, and likely have non-overlapping physiological functions.

## Discussion

We propose that the Hdr:ACDS:Mer complex is a multienzyme “router” that directs substrates and electrons through either the acetyl-CoA or methanogenesis pathways by connecting the CoM-S-S-CoB, acetyl-CoA, and CH_3_-H_4_MPT metabolic nodes (Figure 3c). Despite the importance of several multienzyme complexes in biology (tryptophan synthase, pyruvate carboxylase, polyketide synthases, etc.) it is unusual that acetyl-CoA, a major node involved in carbon fixation, is physically linked with the electron transport system in *Methanosarcina* by the Hdr:ACDS:Mer multienzyme complex [20], [21], [22].

**Figure 4:**
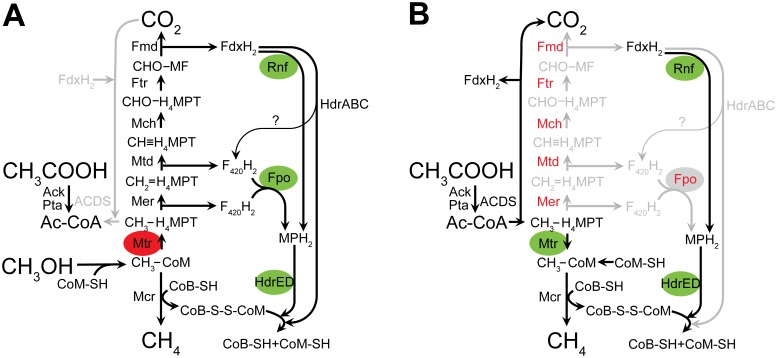
Enzymes used by *M. acetivorans*. Current pathway models for growth of *M. acetivorans* on *A*, methanol + acetate or *B*, acetate as carbon and energy sources. Enzymes in red are essential despite no defined purpose in the pathway. Green ovals = energy conserving step. Red ovals = energy-consuming step. Please see text for abbreviations.

These studies suggest reduction of CoM-S-S-CoB and oxidation of CH_3_-H_4_MPT is physically linked to acetyl-CoA in *M. acetivorans* by a HdrD:ACDS:Mer complex. The HdrD:ACDS:Mer complex we identified, though detected in cells grown on methanol, likely exists in cells grown on acetate because all three enzymes are essential for both the methylotrophic and aceticlastic methanogenesis pathways (Figure 4). Our findings are consistent and complementary to previous reports of CO:Hdr activity in acetate-grown cell extracts from *M. barkeri* and in a reconstituted system using purified components from acetate-grown *M. thermophila* [9], [10]. During methylotrophic growth, ACDS is the enzyme responsible for acetyl-CoA synthesis from CO_2_, CH_3_-H_4_MPT and reduced ferredoxin. During acetoclastic growth, ACDS functions in the opposite direction to cleave acetyl-CoA with the production of CO_2_, reduced ferredoxin, and CH_3_-H_4_MPT, which is reduced to methane [14], [23]. The CdhC β subunit of ACDS houses the NiFe “A site” and catalyzes acetyl-CoA formation from enzyme-bound CO (CO_2_ reduced by ferredoxin) and enzyme-bound methyl-corrin (derived from CH_3_-H_4_MPT) [24]. HdrED, ACDS, and Mer are all essential for methylotrophic and acetoclastic growth, and therefore the HdrD:ACDS:Mer complex likely participates in both methanogenesis pathways.

Redox potentials suggest it is possible that electrons can directly flow from CdhC (−540 mV midpoint potential of the A site of *Clostridia thermoaceticum* ACDS enzyme) to HdrD1 (−142 mV) during acetoclastic growth [25], [26]. However, direct reduction of CoM-S-S-CoB via ACDS:HdrD would bypass Rnf, the proposed sodium-pumping ferredoxin:methanophenazine oxidoreductase, thus preventing formation of a transmembrane ion potential that is necessary for ATP synthesis. During methylotrophic growth ACDS accepts electrons from ferredoxin, and it is possible that HdrD could also compete for electrons from ferredoxin due to its close proximity to CdhC. Such an arrangement would shift flux away from acetyl-CoA biosynthesis towards reduction of CoM-S-S-CoB, albeit at the expense of the ion transmembrane gradient. Genetic and biochemical evidence demonstrates that electrons from ferredoxin are more likely to be oxidized by Rnf and HdrABC. Both HdrABC and Rnf are thought to account for most, but not all, ferredoxin oxidation during methylotrophic methanogenesis (Buan, Kulkarni, Guss, and Metcalf, unpublished data). If HdrD can directly accept electrons from CdhC, or if it competes with CdhC for electrons from ferredoxin, this is expected to be a low-flux pathway that the cell may use to maintain redox balance between ferredoxin, coenzyme A, and CoM-S-S-CoB pools.

If ACDS and Mer conformations are altered as a result of forming a complex in vivo, disruption of the ACDS:Mer interaction is expected to have a negative effect on biosynthesis and oxidative methylotrophic pathways. A recent report by Matschiavelli et al. supports the idea that ACDS and Mer are linked in vivo. The authors showed that deletion of both copies of ACDS results in an increase in the doubling time of *M. acetivorans* by 14 hours during growth on methanol + acetate [14]. The authors hypothesize that the rate of acetyl-CoA production from acetate via Ack and Pta are limiting in the MCD21 (*Δcdh2 Δcdh1* double mutant) and MCD213 (*Δcdh2 Δcdh1 ΔcdhA3* triple mutant) strains. However, deletion of ACDS should not show an effect during growth on methanol + acetate because in wild-type cells, methanol is converted to CO_2_ and methane, while acetate is activated to acetyl-CoA by Ack and Pta, bypassing the need for ACDS. We propose an alternate hypothesis: that flux through the oxidative branch of the methylotrophic pathway is affected due to disruption of the ACDS:Mer complex and a resulting conformational effect on Mer decreases catalytic efficiency of conversion of methyl-H_4_MPT to methylene-H_4_MPT.

Reports of CO:Hdr activity in extracts and a reconstituted system, combined with the observation of unexpected methylotrophic growth phenotypes resulting from ACDS mutations, and of methyl oxidation mutations demonstrating a need for acetate supplementation, supports our hypothesis that the HdrD:ACDS:Mer complex we observed has a physiological role in methanogens. Direct interaction between ACDS and Mer may explain why some methyl-H_4_MPT is oxidized to CO_2_ during growth on acetate [27], and why attempts to delete the oxidative genes in *M. acetivorans* have been unsuccessful. Previous reports suggest the oxidative branch is essential during acetoclastic growth due to the need to generate reduced F_420_ for biosynthetic reactions. In the closely related organism, *M. barkeri*, a *Δmer* mutant is viable but can only grow by the methyl respiration pathway, which *M. acetivorans* cannot use, or mixotrophically on methanol + acetate, albeit very slowly by an unknown Mer/Mtr bypass pathway [28]. Wild-type *M. barkeri* does not grow mixotrophically on methanol + acetate, but uses methanol for methylotrophic methanogenesis, and acetate for biosynthesis. Therefore, in both *M. acetivorans* and *M. barkeri* a *Δmer* mutant cannot use the acetoclastic methanogenesis pathway despite the fact that *M. barkeri* can use hydrogen as an energy source and *M. acetivorans* cannot.

Physical linkage of ACDS and Mer in *M. acetivorans* has intriguing implications as to how carbon flux through the oxidative branch of methanogenesis and biosynthesis pathways in this organism is controlled. ACDS and Mer both use CH_3_-H_4_MPT as substrate, and physically linking these two enzymes means their active sites are in direct competition for substrate. Therefore, as CH_3_-H_4_MPT is produced by Mtr, whether or not it is funneled through the oxidative branch of the methylotrophic methanogenesis pathway is dependent on the rate at which it enters the Mer active site. If Mer is not in a favorable conformation to accept substrate, methyl-H_4_MPT will be available for ACDS to convert into acetyl-CoA for biosynthesis. Furthermore, all the major electron carriers (F_420_, CoM-S-S-CoB, methanophenazine, ferredoxin) converge on the Hdr:ACDS:Mer complex, and phenotypic behavior of ACDS and Mer mutant strains indicates the Hdr:ACDS:Mer complex acts as an integrated switch that samples the redox status of electron carrier pools. The order of substrate and electron donor/acceptor binding determines whether CH_3_-H_4_MPT is fixed as acetyl-CoA by ACDS or is directed to the oxidative branch of the methanogenesis pathway via Mer. By forming a Hdr:ACDS:Mer complex, the cell samples availability of substrates and electron carriers in a minimal spatial location with no need for diffusion of metabolites across cytoplasm. Our data suggests the CH_3_-H_4_MPT metabolite is channeled to one of two metabolic fates (acetyl-CoA production or the oxidative branch of methylotrophic methanogenesis) by a single Hdr:ACDS:Mer protein complex, in contrast to enzyme channeling models that propose an “assembly-line” arrangement of enzyme functions [29], [30].

The 3-dimensional spatial organization of metabolism in methanogens may have evolved as a result of the thermodynamic pressure methanogens face. Methanogens obtain very little ATP/mol substrate consumed (approximately 0.5 ATP/acetate or 1 ATP/MeOH), with only acetogens and syntrophs known to survive under even less thermodynamically favorable conditions. The ability to thrive on so little energy could very well result from exquisitely tight control of substrate and electron channeling that is not necessary in, for instance, a facultative aerobic bacteria like *E. coli* which obtains more energy per mole substrate. Perhaps a fitting analogy would be to describe *E. coli* as a mechanical machine with metabolic “units” that can be interchanged, whereas *Methanosarcina* is a solid-state computer, with a hard-wired multienzyme “biological router” that controls flux through acetyl-CoA as well as through methanogenesis (Figure 1). If multienzyme redox routers exist in other organisms, one would predict they may be found in organisms that also live near the thermodynamic limit of life.

## Materials and Methods

### Growth of *E. coli*

*E. coli* strains were grown in LB medium [31] with the appropriate antibiotics or additions in the following concentrations: rhamnose 5 mM, chloramphenicol 35 or 5 µg/ml.

### Growth of M. acetivorans

*Methanosarcina* strains were grown under strictly anaerobic conditions in HS mineral salts medium [32]. For growth on solid medium, cells were plated on HS medium containing 1.4% agar (w/v) with the appropriate carbon source and additions as previously described [33]. All strains were inoculated into 100 mL of high salt media with a methanol carbon source into 250 mL bottles. The cultures were incubated at 35°C in a Thermo Scientific MaxQ 6000 Incubated/Refrigerated Stackable Shaker until exponential phase. The following anaerobic additions were added when appropriate: MeOH (125 mM), acetate (120 mM), TMA (50 mM), Puromycin (2 µg/ml).

### Strain construction

Genetic methods for *M. acetivorans* are well-defined [34]. Expression of tagged proteins is achieved by creating oligos to amplify the genes of interest and cloning the resultant PCR products into the pJK026A shuttle plasmid at the NdeI and BamHI (or HindIII) restriction sites (Table S6 in File S1). The oligos are designed to fuse the strep-tagII peptide (which has been codon optimized for expression in *M. acetivorans*) to the 5′ or 3′ end of the gene coding sequence [35]. The resulting plasmid (Table 1) is transformed into *M. acetivorans* to create the strains listed in Table 1. Puromycin-resistant colonies are single-colony purified and screened by PCR to ensure the expression plasmid has integrated at the *hpt* locus via ΦC31 integrase [18]. Expression of the tagged protein is driven by the constitutive *P_mcrB_* promoter on pJK026A.

### Strep-tagged affinity co-purification

Protein purification was performed at room temperature under anaerobic conditions. 100 mL cultures were transferred to 15 mL conical tubes and centrifuged at 1228×*g* in a Thermo Scientific IEC Medilite Microcentrifuge for five minutes. The cell pellet was resuspended in 1 mL of 50 mM NaH_2_PO_4_, 0.4 M NaCl, pH 7.2 buffer and transferred into a microfuge tube. 30 mg of DMS cross-linking agent (Dimethyl suberimidate·2 HCl, ThermoFisher Pierce, USA), was added to the remaining cells and mixed for 1 hour. The sample was added to 4 mL of 50 mM TrisCl pH 8 (lysis/quenching buffer). 10 µL of DNase and 50 µL of Halt Protease Inhibitor Cocktail 100X (ThermoFisher Pierce, USA) were added and the sample was incubated for 5 minutes. 100 µL of Streptavidin Agarose Resin (Qiagen, USA) was added, and the sample put on ice for 1 hour with occasional gentle mixing. The sample was placed into 2 mL centrifuge columns and centrifuged for 5 minutes at 1228×*g*. The resin was washed four times with 2 mL 50 mM NaH_2_PO_4_, pH 7.2. Strep-tagged protein was eluted twice with 200 µL biotin eluting buffer (50 mM NaH_2_PO_4_, 300 mM NaCl, 0.05% tween 20, pH 8.0, 10 mM biotin), and the resin was stripped with two washes of 200 µL of 8 M guanidine hydrochloride.

### SDS PAGE and Western blot

Protein concentrations were measured using the Coomassie Plus Bradford Assay (Bio-Rad, USA). Protein samples were mixed with 6X cracking buffer (58 mM Tris pH 6.8, 58 mM SDS, 100 mM dithiothreitol (DTT), 0.68 mM glycerol, 30 µM bromophenol blue) and boiled for 2 minutes. Proteins and Precision Plus Protein Western Standard ladder were separated on precast 4–20% SDS-PAGE gels, and blotted onto ImmunoBlot PVDF membrane (Bio-Rad, USA). Strep-tagged protein was detected with mouse monoclonal anti-StrepTagII antibody (Qiagen, USA) and sheep anti-mouse HRP-linked secondary antibody (GE Healthsciences, USA). HRP signal was detected using the ECL chemiluminescent detection system (ThermoFisher Pierce, USA). Western blot performed with extracts from vector-only control cells did not detect any protein bands.

### Mass spectrometry

For in-solution digests, enriched proteins after elution from the beads were subjected to “shot gun” protein analysis by direct in-solution trypsin digestion of eluent. Eluted protein samples were desalted and dialyzed with 100 mM ammonium bicarbonate using a Millipore Centrifugal Filter Unit (Millipore, USA). Samples were reduced with DTT (7.5 mg), and alkylated with iodoacetamide (0.72 mg) in the dark. Proteins were digested with approximately a 1∶50 trypsin:protein ratio sequencing-grade trypsin (Roche) dissolved in 100 mM ammonium bicarbonate at at 37°C overnight. Tryptic peptides were desalted and concentrated using PepClean C-18 spin columns according to manufacturer’s instructions (Thermo Fisher Scientific, USA).

For in-gel digests, eluted protein was first separated by SDS PAGE and stained with SimplyBlue Safe Stain (Invitrogen). Bands were excised, and gel slices were destained in 1∶1 100 mM ammonium bicarbonate: acetonitrile, and washed in 100% acetonitrile before drying in a speedvac. Tris(2-carboxyethyl)phosphine (TCEP, 100 µl) was added to reduce the protein, and the sample was incubated at 56°C for 45 minutes. Samples were alkylated with iodoacetamide (0.72 mg) and gel slices were washed in 100 mM ammonium bicarbonate. Gel slices were washed twice with 100% acetonitrile and dried in a speed vac. Trypsin was added and gel slices were allowed to swell at 4°C. Gel slices were incubated in 50 mM ammonium bicarbonate at 37°C overnight, and eluted tryptic peptides were desalted as above. Biological replicates of the digested peptide samples were submitted to the University of Nebraska-Lincoln Redox Metabolomics and Proteomics Core Facility.

One dimensional LC-MS/MS was performed with an ultimate 3000 Dionex MDLC system (Dionex Corporation, USA) integrated with a nanospray source and LCQ Fleet Ion Trap mass spectrometer (ThermoFisher Scientific, USA). LC-MS/MS included an on-line sample pre-concentration and desalting using a monolithic C_18_ trap column (Pep Map, 300 µm I.D×5 mm, 100 Å, 5 µm, Dionex, USA). Desalted peptides were eluted and separated on a C_18_ Pico Frit analytical column (75 µm I.D×15 cm, 3 µm, 100 Å, New Objective, USA) by applying an acetonitrile (ACN) gradient (ACN plus 0.1% formic acid, 90 minute gradient at a flow rate of 300 µl/min) and were introduced into the mass spectrometer using the nano spray source. The LCQ Fleet mass spectrometer was operated with the following optimized parameters: nano spray voltage, 2.0 kV; heated capillary temperature, 200°C; full scan *m/z* range, 400–2,000). The mass spectrometer was operated in data dependent mode with 4 MS/MS spectra for every full scan, 5 microscans averaged for full scans and MS/MS scans, a 3 *m/z* isolation width for MS/MS isolations, and 35% collision energy for collision-induced dissociation.

The MS/MS spectra were searched against *M. acetivorans* proteome database using MASCOT (Version 2.2 Matrix Science, London, UK). Database search criteria were as follows: enzyme: trypsin, missed cleavages: 2; mass: monoisotropic; fixed modification: carbamidomethyl (C); variable modification: oxidation (M); peptide tolerance: 1.5 Da; MS/MS fragment ion tolerance: 1 Da. Protein identifications were accepted with a statistically significant MASCOT protein score that corresponds to an error probability of p<0.05. Datasets from duplicate vector only control mock purifications were used as a screen. Raw datasets can be found in Tables S2-S6 in File S1. Protein hits were required to be identified (score >0) in at least two independent purifications for inclusion in Table 2.

MASCOT results were loaded into a MySQL database as a list of identified proteins (nodes) and potential interactions with HdrD for each purification sample. Queries compared samples with the control to identify interactions in both independent purification samples. These results were then visualized using Cytoscape [36].

## Supporting Information

Figure S1 **Analysis of XL-MS results.** Peptide hits from duplicate biological replicates after crosslinking and strep-tag affinity purification were compared. *A*, control protein samples. *B,* samples from cells overexpressing strepHdrD1 protein. blue: hits found only in one control sample, yellow: hits found in both control samples, orange: hits found in one strepHdrD1 sample, red: hits found in both strepHdrD1 samples. Data was visualized using Cytoscape.
(TIF)

File S1 **Supporting tables. Table S1, XL-MS data for control sample 1. Table S2, XL-MS data for control sample 2. Table S3, XL-MS data for strepHdrD1 sample 1. Table S4, XL-MS data for strepHdrD1 sample 2. Table S5, XL-MS data for HdrD2strep sample. Table S6, Oligos used for strain construction.** ****
(XLSX)
