# Supplementary figures and images for: A Multienzyme Complex Channels Substrates and Electrons through Acetyl-CoA and Methane Biosynthesis Pathways in Methanosarcina
Source: PLoS One. 2014 Sep 18;9(9):e107563. doi: 10.1371/journal.pone.0107563 (PMC4169405; doi:10.1371/journal.pone.0107563)

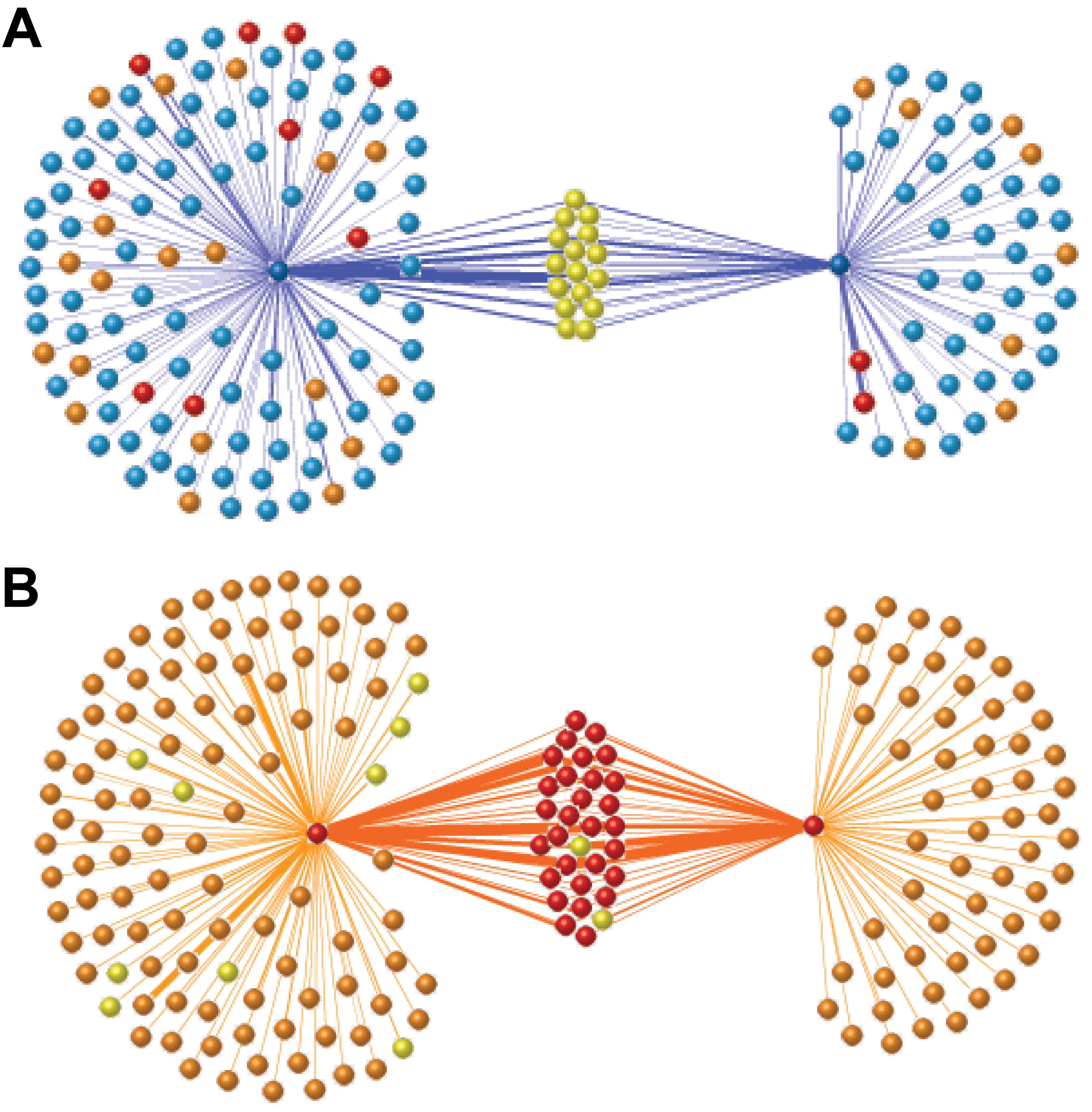

Supplement: Figure S1 — Analysis of XL-MS results. Peptide hits from duplicate biological replicates after crosslinking and strep-tag affinity purification were compared. A, control protein samples. B, samples from cells overexpressing strepHdrD1 protein. blue: hits found only in one control sample, yellow: hits found in both control samples, orange: hits found in one strepHdrD1 sample, red: hits found in both strepHdrD1 samples. Data was visualized using Cytoscape. (TIF) [file pone.0107563.s001.tif]
